# Appraisal of literature reviews on end-of-life care for minority ethnic groups in the UK and a critical comparison with policy recommendations from the UK end-of-life care strategy

**DOI:** 10.1186/1472-6963-11-141

**Published:** 2011-06-02

**Authors:** Natalie Evans, Arantza Meñaca, Erin VW Andrew, Jonathan Koffman, Richard Harding, Irene J Higginson, Robert Pool, Marjolein Gysels

**Affiliations:** 1 Barcelona Centre for International Health Research (CRESIB, Hospital Clínic - Universitat de Barcelona), C/ Rosselló 132 Sobre ático, 08036 Barcelona, Spain; 2 Department of Palliative Care, Policy and Rehabilitation, Cicely Saunders Institute, King's College London, Bessemer Road, London SE5 9PJ, UK; 3 Centre for Global Health and Inequality, University of Amsterdam, O.Z. Achterburgwal 185, 1012DK, Amsterdam, the Netherlands

## Abstract

**Background:**

Evidence of low end-of-life (EoL) care service use by minority ethnic groups in the UK has given rise to a body of research and a number of reviews of the literature. This article aims to review and evaluate literature reviews on minority ethnic groups and EoL care in the UK and assess their suitability as an evidence base for policy.

**Methods:**

Systematic review. Searches were carried out in thirteen electronic databases, eight journals, reference lists, and grey literature. Reviews were included if they concerned minority ethnic groups and EoL care in the UK. Reviews were graded for quality and key themes identified.

**Results:**

Thirteen reviews (2001-2009) met inclusion criteria. Seven took a systematic approach, of which four scored highly for methodological quality (a mean score of six, median seven). The majority of systematic reviews were therefore of a reasonable methodological quality. Most reviews were restricted by ethnic group, aspect of EoL care, or were broader reviews which reported relevant findings. Six key themes were identified.

**Conclusions:**

A number of reviews were systematic and scored highly for methodological quality. These reviews provide a good reflection of the primary evidence and could be used to inform policy. The complexity and inter-relatedness of factors leading to low service use was recognised and reflected in reviews' recommendations for service improvement. Recommendations made in the UK End-of-Life Care Strategy were limited in comparison, and the Strategy's evidence base concerning minority ethnic groups was found to be narrow. Future policy should be embedded strongly in the evidence base to reflect the current literature and minimise bias.

## Background

### Evidence-based public healthcare policy

Public health policy is ideally informed by an up-to-date and unbiased evidence base [1]. This can take the form of research studies, expert opinion, public consultations and literature reviews (systematic or traditional narrative). Many of these sources are potentially subject to various forms of bias. Systematic literature reviews, however, are distinguished from traditional narrative reviews by attempts to minimise bias through the employment of a transparent, rigorous and repeatable review procedure [2,3]. Systematic reviews are becoming increasingly important in healthcare research [1,4] and are particularly useful for policy makers as they not only summarise a large body of literature, but enable novel insights to emerge from the synthesis of multiple studies findings [5]. The systematic review of quantitative studies, particularly randomised controlled trials (RCTs), has come to be seen as a 'gold standard' in healthcare research [2].

Systematic reviews of qualitative studies are, however, also important, as quantitative research can be inadequate for the comprehension of important issues such as understanding patients' healthcare seeking behaviour or the acceptability of interventions [6,7]. These may be better ascertained through thorough, in-depth, qualitative research into practitioners' and patients' attitudes, beliefs, and preferences [6,7]. As such, the systematic review of qualitative literature is also gaining popularity in healthcare research [8].

### End-of-life care: the minority ethnic group experience

The low use of services by members of minority ethnic groups is an issue which is gaining increasing attention from policy makers within end-of-life (EoL) care (the term 'minority ethnic group' is used, in accordance with the official classification used by the UK Office for National Statistics, to describe groups other than 'white British'). First highlighted by Rees [9], in a study of 'immigrant' use of one hospice, the issue did not, however, reach the policy agenda until a decade later, when Hill and Penso [10] drew attention to disparities between minority ethnic groups' estimated need of services and their actual service use. Further research has confirmed low use of EoL care services and identified a number of reasons for this, including: younger population age structure; lower rates of cancer; lower rates of referrals; low awareness of services; and, some culturally inappropriate services [10-18], This growing body of literature has given rise to a number of literature reviews, which have focused on various aspects of EoL care and minority ethnic groups [19-31]. Given the number of reviews and their range of foci it is essential to assess their quality and, therefore, their potential to represent the evidence base and inform policy. A similar approach has been used for the evaluation of literature reviews of palliative care services [32] and interventions to improve care [33].

Various policy initiatives have included commitments to ensure equal access for people from minority ethnic groups to EoL care services [27,34-37]. The most recent of which is the End-of-Life Care Strategy [37], the Department of Health's first comprehensive policy document for EoL care [38]. Some policy documents explicitly state the importance of sensitivity to cultural and religious differences and the need for services to provide 'culturally sensitive' care to reduce inequalities [27,35-37]. Cultural competency training is also identified as a priority for healthcare staff [27,35-37].

### Aim and objectives

This article aims to evaluate the reviews of the literature concerning minority ethnic groups and EoL care in the UK. It is important to critically assess reviews in order to examine their quality, identify gaps in knowledge and assess their suitability as an evidence base for policy.

Specific objectives include: to identify all reviews (systematic and non-systematic) regarding minority ethnic groups and EoL care from the UK; to examine the methodological quality of the reviews; to carry out an interpretive synthesis of reviews' findings; and, to identify recommendations for service improvement.

## Methods

### Review procedure

This work was undertaken as part of the 'PRISMA' programme [39]. 'Reflecting the **P**ositive dive**R**sities of European pr**I**orities for re**S**earch and **M**easurement in end of life c**A**re' (PRISMA) is a three year coordinating action funded by the European Union under the Seventh Framework Programme (FP7) [39]. PRISMA is an integrated programme consisting of eight work packages which aim to co-ordinate research priorities and practice about end-of-life care across Europe and Africa [39]. PRISMA incorporated a work package on the influence of culture on EoL care [39].

### Search strategy

Thirteen electronic databases were searched using the search terms in table 1. In addition, the reference lists of retrieved articles and the archives of key journals (selected if they contained a high number of relevant articles or were medical social science, death and/or palliative care specific) were searched (table 1). Publications by authors of included articles were searched via authors' web pages (when available) and the Web of Knowledge 'author search' facility. In addition, as part of the PRISMA project a network of experts in cultural issues in EoL care was set up. Experts recommended literature, including unpublished and grey literature.

**Table 1:** Databases and hand searches/search terms

Databases (update search to mid Oct 2010)	Search Terms	Hand Search of Journals (update search to mid Oct 2010)
Web of Knowledge all databases (Web of Science with conference Proceedings (1899-2010), BIOSIS Previews (1969-2010), Inspec (1969-2010), MEDLINE (1950-2010), Journal Citation Reports (2000-2010)); OVID (AMED (1985-2010); MEDLINE (1950 to 2010); PsycINFO (1806 to 2010); and EMBASE (1980 to 2010)); Cancerlit (1975-2010); ASSIA (1987-2010); CINAHL (1982 to 2010); and Cochrane reviews (1996-2010).	("United Kingdom" OR UK OR Britain OR England OR Wales OR Scotland OR "Northern Ireland") ***AND*** (palliative OR terminal OR "end of life" OR end-of-life OR death OR dying OR "continu* care" OR "advance directive*" OR hospice* OR "supportive care") ***AND*** (cultur* OR intercultural OR cross-cultural OR transcultural OR ethnic* OR migrant* OR minorit* OR diversity OR Muslim* OR Jew* OR Christian* OR Sikh* OR Buddh* OR Hindu* OR India* OR Pakistan* OR black OR white OR Caribbean* OR Africa* OR Bangladesh* OR Irish OR British OR Chinese OR Asian*)***^a^***	European Journal of Palliative Care 1994-2010 International Journal of Palliative Nursing 1996-2010 Palliative Medicine 1987-2010 Journal of Palliative Care 1985-2010 Diversity in Health and Social Care 2004-2008 (Diversity in Health and Care 2008-2010) Omega 1970-2010 Mortality 1996-2010 Medical Anthropology Volume 21 2002-2010

***^a ^***The official classifications for ethnicity and religious affiliation used by the UK Office of National Statistics, whereas the words 'cultur*, intercultural, cross-cultural, transcultural, ethnic*, migrant*, minorit* and diversity' were chosen in order to retrieve articles concerning cultural competence/sensitivity/humility and minority ethnic groups.

### Inclusion criteria

Reviews were included if they reported on minority ethnic groups and EoL care in the United Kingdom (UK). Only literature reviews were included; a systematic review of primary research has been undertaken as a second part of the project and published elsewhere [40]. No relevant foreign language reviews were identified.

Both traditional (non-systematic) and systematic reviews were included. Non-systematic reviews were included, despite difficulties in assessing both their methodological quality and their propensity for bias, because the use of systematic search procedures is a relatively recent development, especially for qualitative and mixed method research. To thoroughly appraise all reviews of the literature on minority ethnic groups, which have potentially influenced British policy, the inclusion of non-systematic reviews was necessary.

### Screening and data extraction

Articles were managed in Endnote X2. Titles and abstracts were initially assessed by one reviewer (NE) to eliminate those not related to EoL care. All remaining titles and abstracts were then assessed for relevance in regular team meetings (minimum three participants). When there was insufficient information to decide upon inclusion, the full text was retrieved and appraised. Data were extracted from full text copies of the reviews (tables 2 and 3) by one reviewer (NE) and checked by another (MG).

**Table 2:** Reviews that met inclusion criteria

Reference	Objective	Methods	Number of articles included	Quality
Ahmed, et al. (2004)[21]	To determine problems and issues in accessing specialist palliative care.	Systematic review (1997-2003). Papers from non-UK sources included.	40	9
Bager, et al. (2009)[29]	To summarises the current research evidence on cultural issues relating to ethnicity in EoL care in care homes.	Non-systematic (narrative) review.	44	N/A
Cox, et al. (2003)[22]	To consider the implications of culture on do-not-resuscitate decision-making and make recommendations for practice.	Systematic. Papers from non-UK sources included.	34	4
Eklan, et al. (2007)[19]	To explore the qualitative literature concerning the experiences of cancer service users from minority ethnic groups.	'Critical'* review (1995-2005). Non-EoL articles included.	25 (11 on EoL care)	7
Firth (2001)[25]	To review the literature concerning minority ethnic groups and EoL care.	Non-systematic (narrative) review (1995-2001). Non-UK and non-EoL articles included.	406	N/A
Gunaratnam, (2006)[28]	To draw attention to the palliative care needs and experiences of elders from minority ethnic groups.	Non-systematic (narrative) review. Papers from non-UK sources included.	53	N/A
House of Commons Health Committee (2004)[27]	To examine the extent to which the needs and wishes of patients of different ages are taken into account, including their care choices, ethnicity, cultural and spiritual beliefs.	Report of the House of Commons fourth session on palliative care.	65 (plus 20 oral statements) (8 pieces of evidence on minority ethnic groups; 4 written and 4 oral)	N/A
Johnson (2001)[26]	To review the literature on palliative care, cancer and minority ethnic communities.	Non-systematic (narrative) review (papers from a broader systematic review[47] included). Papers from non-UK sources included.	12 (3 on EoL care)	N/A
Jones (2005)[20]	To explore the qualitative literature concerning EoL issues and ethnicity/race/diversity.	Systematic review. Papers from non-UK sources included.	119	4
Payne, et al. (2005)[24]	To explore Chinese cultural perspectives on EoL care.	Systematic review. Papers from non-UK sources included.	10	5
Redman, et al. (2008)[23]	To explore the evidence concerning race, ethnicity, cancer and cancer services	'Critical'* review.	31 (eight on EoL care)	7
The Department of Health (2008)[30]	To provide evidence concerning the diversity of EoL experiences.	Non-systematic (narrative) review and public consultation.	23 (5 on 'race' and 2 on 'religion and belief')	N/A
Walshe, et al. (2009)[31]	To identify whether patients with different characteristics use community palliative care services in different ways.	Systematic (1997-2008). Papers from non-UK sources included.	48	8

*Critical reviews took a systematic approach to the search procedure but reported only on selection of the articles retrieved.

**Table 3:** Grading of methodological quality of the systematic and critical reviews

Criteria	Components	Scores	Agreed Scores for Each Review						
			Ahmed (2004) [21]	Cox et al. (2006) [22]	Elkan, et al. (2007) [19]	Jones (2005) [20]	Payne, et al. (2005) [24]	Redman, et al. (2008) [23]	Walshe, et al. (2009) [31]
Specifying the objectives		precise = 2 vague = 1 implicit = 0	2	2	2	2	2	2	2
Searching the literature	Electronic databases, journal searches, grey literature, reference lists, unpublished sources known to experts (via personal communication)[42], author searches.	4+methods = 2 2 or 3 = 1 0 or 1 = 0	1	2	1	1	1	0	1
Selecting relevant and valid studies	Search terms specified, inclusion/exclusion criteria specified, studies chosen relevant to research question[2], 2+ reviewers.	4+methods = 2 2 or 3 = 1 0 or 1 = 0	2	0	1	0	1	1	1
Critical appraisal of studies	Data extraction categories relevant to research question, studies graded (or grading explicitly rejected)*.	both = 2 only one = 1 implicit = 0	2	0	1	0	0	2	2
Synthesis of data and presentation of findings	Table of included studies, discussion of methodological quality of studies, rigorous qualitative overview or meta-analysis (rigorous or rejected), limitations, implications for health care, implications for research.	4+ components = 2 2 or 3 = 1 0 or 1 = 0	2	0	2	1	1	2	2
**Total Score**			**9**	**4**	**7**	**4**	**5**	**7**	**8**

*As the grading of qualitative studies is controversial [43,44], an explicit rejection of grading, with justification, was accepted.

### Analysis

#### Assessment of methodological quality

Reviews that took a systematic approach were appraised for methodological quality (table 3) using a grading scheme adapted from Russell, et al. [41] and influenced by Hawker, et al. [2], Greenhalgh [42], Goldsmith, et al. [43] and Mays, et al. [44]. Five areas of reporting were graded: specifying the objectives; searching the literature; selecting relevant and valid studies; critical appraisal of studies; and, synthesis of data and presentation of findings (more details in table 3). Reviews were graded independently by two reviewers (NE and MG) and then compared. Differences were discussed in team meetings (minimum 3 people) and resolved by consensus. It was only possible to grade the methodological quality of reviews that had followed a systematic search procedure.

No studies were excluded on the basis of quality, as it was not possible to grade non-systematic reviews. In addition, there is no agreement on the role of quality criteria especially when reviews include qualitative and mixed methods studies. Instead, for transparency, it has been made explicit which reviews are systematic and which are non-systematic. Furthermore, the difference in quality of systematic reviews is highlighted in the score breakdown (table 3).

#### Identification of key themes

An interpretative approach, following the principles of constant comparison was used to identify key themes from the reviews [45,46]. The findings from each review were coded, categorised and summarised. Codes and categories were iteratively compared and contrasted, cross-cutting themes were identified, which were reduced to a number of key themes [45,46]. Finally, a narrative synthesis of findings concerning the key themes was conducted, paying particular attention to commonalities and discordance between reviews.

This article does not intend, however, that a review of reviews should be used as an evidence base for policy. Such an approach would presuppose that those who conducted the original reviews were correct in both their approach and assessments of the evidence. In contrast, the purpose of this synthesis is to provide a representation of the findings of existing reviews of the literature.

#### Results

**Figure 1:**
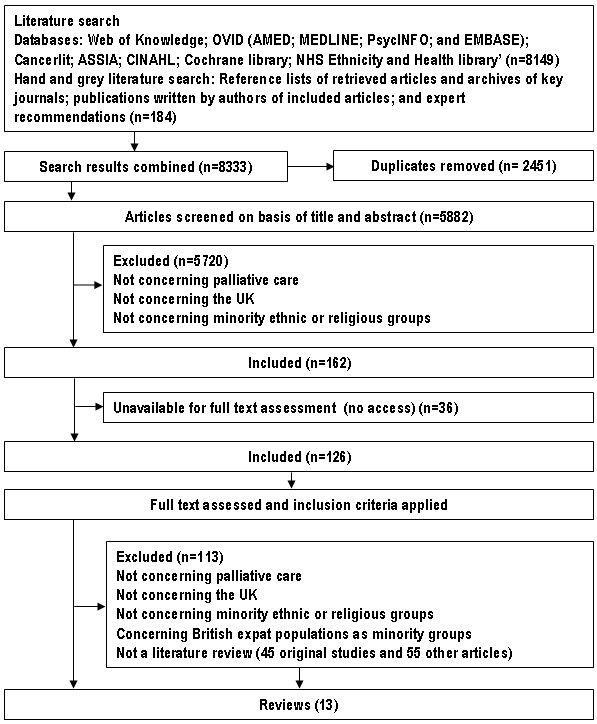
**Flow diagram detailing the article selection process**.

After removing duplicates, a total of 5882 citations were screened. Of these, 5720 were discarded after reviewing the title and abstract as they did not fulfil the inclusion criteria. Thirty-six articles were not available for full text assessment (none of these were reviews). The full text of 126 articles was examined in more detail. On closer examination, 13 articles were found not to meet inclusion criteria and a further 100 were not reviews (45 original studies and 55 other articles). A total of 13 reviews met inclusion criteria (Figure 1), details of reviews [19-31] and related articles [47] can be seen in table 2.

A number of included reviews had a narrow focus; restricting the review by ethnic group [24], a particular aspect of EoL care [21,22], or EoL care setting [29,31]. In addition, broader reviews of cancer services and minority ethnic groups were included if they reported significant findings regarding EoL care [19,23]. All reviews were published between 2001 and 2009 (table 2).

Reviews' inclusion criteria differed in line with their varied focus. In addition, five reviews only included primary research [19,21,23,24,31], whereas eight reviews [20,22,25-30] included overviews, opinion pieces, and even web-based resources to build a picture of minority ethnic groups' experience of EoL care. The majority of the articles included in the reviews came from the UK; five reviews [19,23,27,29,30] included evidence from the UK only, whereas eight reviews included non-UK sources to a greater or lesser extent [20-22,24-26,28,31]. Where reviews included material from non-UK sources this has been highlighted in table 2. Due to the difference in reviews' aim, scope and inclusion criteria it is difficult to judge the success or the relevance of a review by the number of articles included alone.

### Methodological quality

Descriptions of the review process ranged from no information to thorough outlines of a systematic review procedure. Seven reviews followed a systematic search procedure [19-24,31]. These reviews were graded for methodological quality according to the criteria in table 3. Quality scores ranged from four to nine out of ten. The mean score was six (median seven), indicating that the 'systematic' reviews were, on average, of a reasonable methodological quality (table 3). These reviews all included electronic database searches and most included reference list searches [19-24,31]. Three reviews included journal hand-searches [21,22,31], one searched grey literature [20], and one contacted experts in the field [19]. The number of reviewers was rarely explicit. Three reviews graded studies for quality [21,23,31]. Four 'systematic' reviews did not grade articles or explicitly rejected grading [19,20,22,24]. The synthesis of data and presentation of findings was found to be good in most reviews (table 3).

The remaining six reviews did not provide sufficient information to allow the grading of quality [25-30]. A lack of detail does not necessarily mean the review process was of poor quality or that results are less significant. It is not, however, possible to assess the rigour of the review process.

### Key themes and findings

An interpretative synthesis of review findings resulted in the following six themes: structural inequality; inequality by disease group; referrals; place of care and death; awareness and communication issues; and, cultural competency.

#### Structural inequality

Structural inequalities which people from minority ethnic groups face due to socio-economic and geographic disadvantages was a reoccurring theme [21,24,28,48]. Services were acknowledged to be 'disproportionally needed in areas of social deprivation, and disproportionally present in areas of social affluence' [27]. Inequality in provision is partly due to the predominately charitable nature of service development; with more donations received in wealthier areas [28,48]. Minority ethnic groups, therefore, face 'double discrimination', as higher concentrations of such groups are found in areas of social deprivation, and even within these areas they have low service use in comparison to 'white British' service users [27]. Elderly members of minority ethnic groups were said to be particularly vulnerable due to the combined effects of low socio-economic status and discrimination [28,29]. Furthermore, carers reported additional problems, such as anxieties regarding housing and visas [23,30].

#### Inequality by disease group

Differences in cancer incidence and mortality were highlighted in a number of reviews [19,20,23,25-28] and the focus on cancer in EoL care provision was cited as a major source of inequality due to the greater importance of non-malignant diseases among minority ethnic groups [20,21,25,30]. Limited service provision for non-malignant diseases was attributed to: uncertain disease trajectories; limited service capacity; different expectations of carers and patients; and, a lack of appropriate expertise [21]. The reviews that focused only on cancer services, however, highlighted that as age structures and lifestyles converge, cancer incidence and mortality among minority ethnic groups can be expected to increase [23,26,28].

#### Referrals

Patients from minority ethnic groups were said to lack 'timely referrals' to specialist EoL care services [21,23,28,31]. Reasons included a limited knowledge of services and the referral process among physicians who are not EoL care or cancer specialists [21]. Physicians were said to resist sharing patients and to perceive institutional care as inappropriate for minority ethnic groups, who prefer to 'look after their own' [21,29,31]. General practitioners were also seen as 'gate-keeping' services through the referral process and poor communication with general practitioners could limit access [28]. Furthermore, some patients rejected referral due to negative perceptions of services [21].

#### Place of death and care

Recent policy initiatives have promoted home deaths on the basis of a reported preference among patients to die at home [27,30]. A number of reviews highlighted the perceived preference among minority ethnic groups for home care [21,23,29]. Payne et al. [24], however, found that the preferred place of death for people from the Chinese community was dependent on multiple factors, including the quality of housing and the length of time spent in housing, and that services could be acceptable when well-established and understood by members of the Chinese community [24]. In contrast, Walshe et al. [31] emphasised that different ethnic groups could have different perceptions of hospice care by highlighting a study in which 'those of Chinese origin living in the UK' were said to perceive hospice care negatively [31]. Above all, the need for discussion and choice regarding place of death was identified as a priority by members of minority ethnic groups [30].

#### Awareness and communication

Lack of information in an appropriate format, negative perceptions and low awareness of services were identified as barriers to service use [19,25,28]. Problems persisted once services had been accessed; poor communication between patients and their families and healthcare professionals was an issue repeatedly emphasised [20-22,25,26,28,48]. Four reviews emphasised the importance of good communication above all other factors [19,22,26]. Specific problems included: a lack of information provided in appropriate languages and formats [19,25,28]; inadequate interpreting and advocacy provision [19,25,28]; differences in social taboos about death and illness [22,25,28]; and, difficulties in understanding and communicating both verbally and non-verbally [22,49]. Due to limited resources, family members were used to interpret information of a sensitive or unsuitable nature, increasing the risk of non-disclosure [25]. Jones [20], however, called for understanding in regard to disclosure and involvement of family members in decision-making. Furthermore, Payne et al. [24] found that most Chinese patients viewed family members as having an important collaborative role in EoL decision-making.

The communication of patients' wishes through advance care planning was discussed just twice in the reviews. Payne et al. [24] found that Chinese patients were more likely to prefer life sustaining treatments and less likely to desire euthanasia. Similarly, Cox et al. [22] emphasised the effects of cultural background on decision-making.

#### Cultural competency

Negative perceptions towards services were considered significant impediments to increased utilisation, and some services were identified as culturally insensitive [48]. A common recommendation was the need for training in care that is sensitive to cultural difference [23-25,28,29,48]. Only one review, however, explicitly defined the terms used for such care ('cultural competence' and 'cultural safety') [25]. In contrast to the frequent recognition that services need to provide culturally competent care, few reviews provided recommendations about how to achieve this. Only one review highlighted the large numbers of minority ethnic staff working in the healthcare services [29]. Cultural differences were said to lead to uncertainty, even when staff were trained in 'cultural competency' issues [29].

Concerns were raised about deterministic links between cultural, ethnic or religious factors and EoL preferences (an approach referred to as the 'cookbook' approach) [20] and the stereotyping of patients was warned against [19,20]. Jones [20] stated that the majority of literature regarding healthcare and minority ethnic groups merely presents information about religious rituals and beliefs. Whereas, Payne et al. [24], in an exploration of Chinese cultural perspectives on EoL care, found that there was little evidence to support Chinese stereotypes. These concerns led authors to emphasize that meeting 'cultural' needs is only part of meeting patients' individual needs [19,20,24,28,30]. In contrast, Cox et al. [22] stated that becoming 'aware' of attitudes, values, beliefs and cultural norms can improve minority ethnic groups' involvement in decision-making. The importance of monitoring service users' ethnicity was frequently stressed [20,23,25,29-31]. However, data was said to be inadequately collected and rarely used to influence service provision [25,29].

### Recommendations for service improvement

A number of recommendations were made in the reviews for improving EoL care services for minority ethnic groups (table 4).

**Table 4:** Recommendations for improving services made in the reviews

○ Strategic planning of services to ensure equity of provision [27,28]
○ End-of-life care provision for non-malignant diseases [21,25]
○ Training regarding services and the referrals pathway for physicians who are not palliative care or cancer specialists [21,26-28]
○ Recruitment of staff from minority ethnic groups and the implementation of equal opportunity policies [21,25,29]
○ Provision of interpretation and advocacy services [25-28,30]*
○ Training of interpreters and advocates in EoL care issues [25]
○ Awareness raising of services among minority ethnic groups using appropriate methods [21,28,30]*
○ Provision of information concerning services in appropriate languages and formats [25-28]
○ Discussion of place of death preferences [25,30]*
○ Understanding that a preference for home care should not be assumed and that all options must be explained [29]
○ Support for carers [25]
○ Understanding of the EoL care needs in care homes [28,29]
○ Attendance of religious and spiritual needs, preferably by a multi-faith chaplaincy service [25]
○ The provision of space and time for religious practices to be carried out [30]*
○ The provision of special dietary requirements on a case by case basis [25]
○ Make care homes and hospices more welcoming [29,30]*
○ Involvement of minority ethnic groups in the planning of services and outreach [23,25,28]
○ Recognition that categorising people by ethnicity alone can lead to stereotyping [20,24,30]*
○ Recognition that cultural needs form only one part of an individual's EoL needs [19,20,24,28,30]*
○ Sensitivity regarding the involvement of patients' families in decision-making and disclosure [19,20,22,25]
○ Training in care that is sensitive to cultural difference [23-25,28,29,48]
○ Training in: communication issues (verbal and non-verbal) [19,24-26]; the use of interpreters [25]; awareness of the multiple disadvantages faced by minority ethnic groups [20,24,28]; information concerning 'attitudes, values, beliefs and norms' of minority ethnic groups [22]; and, countering the belief that services are unsuitable for minority ethnic groups [21,23]
○ Training at under-graduate and post-graduate level [25] and to both generalists and specialists [27]
○ Extra funding for training, interpretation and awareness raising [28]
○ Rigorous ethnic monitoring of service users and services reviewed using data [20,23,25,26,29,30]*
○ Tackling of racism[19,23,25,28]

* Recommendations made in the End-of-Life Care Strategy: Equality Impact Assessment [30].

## Discussion

The literature reviews concerning minority ethnic groups and EoL care in the UK described a range of social, institutional, epidemiological and cultural reasons for low service use and identified some distinct EoL preferences and needs. In light of this evidence, in order to improve the use of, and satisfaction with, palliative care services by such groups, it is necessary to recognise the complexity of factors leading to low service use and sub-standard provision of care and implement a systematic, organisation wide, approach to tackling these multiple and inter-related factors. The influence of these multiple and inter-related factors was reflected in the reviews' recommendations for service improvement (table 4).

Of the thirteen reviews identified, seven took a systematic approach [19-24,31], and four scored highly for methodological quality [19,21,23,50]. These reviews provide a good reflection of the evidence base and could be used to inform policy. Six reviews did not provide sufficient information for their quality to be graded, including the End-of-Life Care Strategy: Equality Impact Assessment [30]. The aim of this document was to ensure that the UK End-of-Life Care Strategy [37] 'does not inadvertently create inequality', but referenced just seven articles concerning 'race' and 'religion'.

Due to the relatively small number of articles included in the End-of-Life Care Strategy: Equality Impact Assessment [30], the themes and recommendations identified from the literature reviews were compared and contrasted with those of the End-of-Life Care Strategy [37].

The recommendations regarding minority ethnic groups made within the Strategy (table 5) were more limited than those made within the reviews (table 4), and omitted a number of recommendations made in its own Equality Impact Assessment [30] (table 4). The recommendations focused on problems that arise during the physician-patient encounter (such as issues regarding communication and awareness of different EoL preferences) or raising awareness of services amongst minority ethnic groups (table 5).

**Table 5:** Recommendations for service improvement from the End-of-Life Care Strategy [37]

○ Commitment to equal access to services
○ Recognition of distinct preferences regarding: the chaplaincy service; support needs of carers and families; organ donation; care and disposal of the corpse; and, bereavement care
○ The holistic assessment of needs, includes spiritual and cultural needs
○ Awareness raising about death and dying in 'religious organisations such as churches, mosques, synagogues'
○ The need for interpretation services
○ The need for the ethnicity and religion monitoring
○ The need for 'spiritual, religious and cultural care competences' to be 'adopted within all core training'

Although inequalities in service provision due to geographical factors or disease group were addressed in the Strategy, their contribution to low service use by minority ethnic groups was not recognised. In addition, a lack of timely referrals for members of minority ethnic groups was not mentioned.

No mention was made to minority ethnic groups in the Strategy's sections on 'place of death', 'core principles and competencies', 'education, training and continued development', 'improving the environment', 'prisons and secure units' (even though one third of prisoners come from minority ethnic groups [51]) and 'future research'.

The need for 'spiritual, religious and cultural care competences' to be 'adopted within all core training' was highlighted, however, these needs were not repeated in the Strategy's sections regarding 'core principles and competencies' and 'education, training and continued development'. In addition, the Strategy took an apparent 'cookbook' approach regarding organ donation, providing the link to a website on 'the perspectives on organ donation of the six major religions in the UK' [37], an approach criticised within the reviews.

In both the End-of-Life Care Strategy [37] and the End-of-Life Care Strategy: Equality Impact Assessment [30] the terms used to describe people from minority ethnic groups were questionable. A case study which described an 'engagement project' referred to the establishment of a dialogue between the hospice and 'ethnic communities' [37]. Use of the term 'ethnic' to describe 'minority ethnic groups' implies that 'ethnicity' is something that only 'minority ethnic groups' have, rather than recognising that, in fact, 'ethnicity' is something that everyone has. Furthermore, the End-of-Life Care Strategy: Equality Impact Assessment [30] used the terms 'minority ethnic', 'black and minority ethnic' and 'race' interchangeably, implying equivalence.

The evidence base which informed the Strategy's recommendations on minority ethnic groups was found to be small. The bibliography includes just one review (Cox et al. [22], which was restricted to looking at minority ethnic groups' involvement in advance care planning). Moreover, just five studies and one report concerning minority ethnic groups were cited [18,22,28,52-55]. The End-of-Life Care Strategy: Equality Impact Assessment [30] did not cite any reviews. Its sections on 'race' and 'religion', however, highlighted a paucity of research looking at inequality due to 'racial' or 'religious' factors and referenced just seven articles [18,52,54,56-59], only five of which were original studies [30].

## Future research

Thirteen reviews concerning minority ethnic groups and EoL care in the UK were identified, of which seven took a systematic approach [19-24,31]. However, the reviews identified either had a narrow focus, such as restricting the review by ethnic group [24] or a particular aspect of EoL care [21,22,31], or were broader reviews of cancer services and minority ethnic groups that reported significant findings regarding EoL care [19,23]. Only one systematic review specifically focused on minority ethnic groups and EoL care in the UK [20]. However, this review received a low quality score (4) and most articles included came from outside the UK. There remains a need for a thorough systematic review of EoL care and minority ethnic groups in the UK to improve the evidence base on which policy initiatives are ideally based.

## Conclusions

Thirteen reviews of the literature concerning minority ethnic groups and EoL care were identified. A number of reviews were systematic and scored highly for methodological quality. These reviews provide a good reflection of the primary evidence and could be used to inform policy. The complexity of inter-related social, institutional, epidemiological and cultural factors leading to low service use were recognised and this complexity was reflected in the reviews' recommendations for service improvement. Recommendations made in the End-of-Life Care Strategy were limited in comparison. However, despite certain omissions, the recommendations made within the Strategy give the impression that minority ethnic groups had been taken into consideration. The lack of integration, however, of these recommendations into key sections of the document brings their real impact into question. In addition, the evidence base, on which recommendations were made, was found to be narrow and some important issues addressed in the Equality Impact Assessment were not addressed.

Public healthcare policy is ideally based upon systematic reviews of the literature. All but three of the thirteen reviews of the literature identified [23,29,31] had been published prior to the publication of the End-of-Life Care Strategy. In response to the finding that these reviews had a minimal influence on the End-of-Life Care Strategy, it is recommended that future policy be based upon systematic reviews of the current literature, or at least upon existing systematic reviews, in order to reflect the current evidence and minimise bias.

## Limitations

Six non-systematic (narrative) reviews (which are more at risk of selection bias) were included. Furthermore, publication bias towards quantitative studies whose results are statistically significant, and towards qualitative studies which have striking or easily understandable findings, may introduce sources of bias into systematic reviews of the literature. The reviews which took a systematic approach were of varying quality, although on average they were of a reasonable standard. The narrow focus of some reviews may have biased results towards these topics.

## Competing interests

The authors declare that they have no competing interests.

## Authors' contributions

NE designed the search strategy, screened articles, carried out data extraction, graded papers, participated in team meetings in which grading of papers was discussed, analysed results and drafted the manuscript. AM participated in team meetings in which grading of papers was discussed and helped to draft the manuscript. EA participated in team meetings in which grading of papers was discussed and helped to draft the manuscript. JK provided help with the search strategy and helped to draft the manuscript. RH participated in its design and coordination of the project and helped to draft the manuscript. IH participated in its design and coordination of the project and helped to draft the manuscript. RP participated in its design and coordination of the project and helped to draft the manuscript. MG screened articles, carried out data extraction, graded papers, analysed findings, participated in team meetings in which grading of papers was discussed, conceived the project and helped to draft the manuscript. All authors read and approved the final manuscript.

## Pre-publication history

The pre-publication history for this paper can be accessed here:

http://www.biomedcentral.com/1472-6963/11/141/prepub

## References

[B1] Brealey S What is evidence based medicine? An emerging science not fashionable rhetoric Radiography 2001 7 1 7 10 10.1053/radi.2000.0296

[B2] Hawker S Payne S Kerr C Hardey M Powell J Appraising the evidence: reviewing disparate data systematically Qualitative Health Research 2002 12 9 1284 10.1177/1049732302238251 12448672

[B3] Cook D Mulrow C Haynes R Systematic reviews: synthesis of best evidence for clinical decisions Annals of Internal Medicine 1997 126 5 376 9054282 10.7326/0003-4819-126-5-199703010-00006

[B4] Bero L Jadad A How consumers and policymakers can use systematic reviews for decision making Annals of Internal Medicine 1997 127 1 37 9214251 10.7326/0003-4819-127-1-199707010-00007

[B5] Walsh D Downe S Meta-synthesis method for qualitative research: a literature review Journal of Advanced Nursing 2005 50 2 204 211 10.1111/j.1365-2648.2005.03380.x 15788085

[B6] Stern J Simes R Publication bias: evidence of delayed publication in a cohort study of clinical research projects British Medical Journal 1997 315 7109 640 9310565 10.1136/bmj.315.7109.640 PMC2127436

[B7] Popay J Rogers A Williams G Rationale and standards for the systematic review of qualitative literature in health services research Qualitative Health Research 1998 8 3 341 10.1177/104973239800800305 10558335

[B8] Lavis J How Can We Support the Use of Systematic Reviews in Policymaking? PLoS Med 2009 6 11 10.1371/journal.pmed.1000141 PMC2777391 19936223

[B9] Rees W Immigrants and the hospice Health Trends 1986 18 4 89 91 10280088

[B10] Hill D Penso D Opening Doors: Improving Access to Hospice and Specialist Palliative Care Services by Members of the Black and Ethnic Minority Communities 1995 National Council for Hospice and Specialist Palliative Care Services PMC2149863 8782800

[B11] Fountain A Ethnic minorities and palliative care in Derby Palliative Medicine 1999 13 2 161 162 10.1191/026921699669352140 10474700

[B12] Bestall JC Ahmed N Ahmedzai SH Payne SA Noble B Clark D Access and referral to specialist palliative care: patients' and professionals' experiences Int J Palliat Nurs 2004 10 8 381 389 15365492 10.12968/ijpn.2004.10.8.15874

[B13] Karim K Bailey M Tunna K Nonwhite ethnicity and the provision of specialist palliative care services: factors affecting doctors' referral patterns Palliative Medicine 2000 14 6 471 10.1191/026921600701536390 11219877

[B14] Simmonds R Improving Access to Palliative Care Services for Ethnic Minority Groups 2001 National Council for Hospice and Specialist Palliative Care Services, London, UK

[B15] Webb L No exclusion clause project, exploring palliative care in Warwickshire 2001 National Council for Hospice and Specialist Palliative Care Services, London, UK

[B16] Randhawa G Owens A The meanings of cancer and perceptions of cancer services among South Asians in Luton, UK British Journal of Cancer 2004 91 62 68 10.1038/sj.bjc.6601892 15162147 PMC2364764

[B17] Koffman J Burke G Dias A Raval B Byrne J Gonzales J Daniels C Demographic factors and awareness of palliative care and related services Palliat Med 2007 21 2 145 153 10.1177/0269216306074639 17344263

[B18] Koffman J Higginson IJ Dying to be home? Preferred location of death of first-generation black Caribbean and native-born white patients in the United Kingdom J Palliat Med 2004 7 5 628 636 10.1089/jpm.2004.7.628 15588353

[B19] Elkan R Avis M Cox K Wilson E Patel S Miller S Deepak N Edwards C Staniszewska S Kai J The reported views and experiences of cancer service users from minority ethnic groups: a critical review of the literature European Journal of Cancer Care 2007 16 2 109 121 10.1111/j.1365-2354.2006.00726.x 17371419

[B20] Jones K Diversities in approach to end-of-life: A view from Britain of the qualitative literature Journal of Research in Nursing 2005 10 4 431 10.1177/174498710501000406

[B21] Ahmed N Bestall J Ahmedzai S Payne S Clark D Noble B Systematic review of the problems and issues of accessing specialist palliative care by patients, carers and health and social care professionals Palliative Medicine 2004 18 6 525 10.1191/0269216304pm921oa 15453624

[B22] Cox C Cole E Reynolds T Wandrag M Breckenridge S Dingle M Implications of Cultural Diversity in Do Not Attempt Resuscitation (DNAR) Decision Making Journal of Multicultural Nursing and Health 2006 12 1 20

[B23] Redman J Higginbottom G Massey M Critical review of literature on ethnicity and health in relation to cancer and palliative care in the United Kingdom Diversity in Health and Social Care 2008 5 2 137 150

[B24] Payne S Chapman A Holloway M Seymour JE Chau R Chinese community views: Promoting cultural competence in palliative care Journal of Palliative Care 2005 21 2 111 116 16114810

[B25] Firth S Wider Horizons: Care of the Dying in a Multicultural Society 2001 National Council for Hospice and Specialist Palliative Care Services

[B26] Johnson M Palliative Care, Cancer and Minority Ethnic Communities: A Literature Review 2001 Leicester: Mary Seacole Research Institute, De Montford University

[B27] House of Commons Health Committee Palliative Care Fourth Report of the Session 2004 1

[B28] Gunaratnam Y Ethnicity, Older People and Palliative Care Joint report from the National Council for Palliative Care and the Policy Research Institute on Ageing and Ethnicity London: National Council for Palliative Care 2006

[B29] Badger F Pumphrey R Clarke L Clifford C Gill P Greenfield S Jackson K The role of ethnicity in end-of-life care in care homes for older people in the UK: a literature review Diversity in Health and Care 2009 6 1 23 29

[B30] DoH End of Life Care Strategy: Promoting High Quality Care for All Adults at the End of Life. Equality Impact Assessment 2008 Department of Health

[B31] Walshe C Todd C Caress A Chew-Graham C Patterns of access to community palliative care services: a literature review J Pain Symptom Manage 2009 37 5 884 912 10.1016/j.jpainsymman.2008.05.004 19097748

[B32] Goodwin D Higginson I Edwards A Finlay I Cook A Hood K Douglas H Normand C An evaluation of systematic reviews of palliative care services Journal of palliative care 2002 18 2 77 12164104

[B33] Grimshaw J Shirran L Thomas R Mowatt G Fraser C Bero L Grilli R Harvey E Oxman A O'Brien M Changing provider behavior: an overview of systematic reviews of interventions Medical Care 2001 39 8 11583120

[B34] Executive N The NHS cancer plan A plan for investment A plan for reform Leeds: Department of Health, NHS Executive 2000

[B35] Improving Supportive and Palliative Care for Adults with Cancer http://www.nice.org.uk/nicemedia/pdf/csgspmanual.pdf

[B36] DoH Cancer Reform Strategy 2007

[B37] DoH End of Life Care Strategy: Promoting High Quality Care for All Adults at the End of Life 2008 Department of Health

[B38] Ingleton C Gott M Kirk S Editorial: The beginning of the end (of life care strategy) Journal of Clinical Nursing 2009 18 7 935 10.1111/j.1365-2702.2008.02616.x 19284430

[B39] Harding R Higginson I PRISMA: A pan-European Co-ordinating action to advance the science in end of life cancer care European Journal of Cancer Care 2010 in press 10.1016/j.ejca.2010.01.035 20185295

[B40] Evans N Meñaca A Andrew EVW Koffman J Harding R Higginson I Pool R Gysels M PRISMA obo Systematic Review of the Primary Research on Minority Ethnic Groups and End-of-Life Care from the UK Journal of Pain and Symptom Management 2011 in press 10.1016/j.jpainsymman.2011.04.012 22001070

[B41] Russell I Di Blasi Z Lambert M Russell D Systematic reviews and meta-analyses: opportunities and threats Evidence-based fertility treatment 1998 15 64

[B42] Greenhalgh T How to read a paper: papers that summarise other papers (systematic reviews and meta-analyses) British Medical Journal 1997 315 7109 672 9310574 10.1136/bmj.315.7109.672 PMC2127461

[B43] Goldsmith M Bankhead C Austoker J Synthesising quantitative and qualitative research in evidence-based patient information British Medical Journal 2007 61 3 262 10.1136/jech.2006.046110 PMC2652927 17325406

[B44] Mays N Pope C Popay J Systematically reviewing qualitative and quantitative evidence to inform management and policy-making in the health field Journal of health services research & policy 2005 10 Supplement 1 6 10.1258/1355819054308576 16053580

[B45] Glaser BG Strauss AL The discovery of grounded theory: Strategies for qualitative research 2007 Aldine Transaction

[B46] Glaser BG The constant comparative method of qualitative analysis Social problems 1965 12 4 436 445 10.1525/sp.1965.12.4.03a00070

[B47] Atkinson M Clark M Clay D Johnson M Owen D Szczepura A Systematic review of ethnicity and health service access for London Primary care 2001 3 3

[B48] Palliative Care Fourth Report of the Session 2004 1 House of Commons

[B49] Firth S Oliviere D, Monroe B Minority ethnic communities and religious groups Death, dying and social differences 2004 Oxford: Oxford University Press

[B50] Walshe C Chew-Graham C Todd C Caress A What influences referrals within community palliative care services? A qualitative case study Social Science & Medicine 2008 67 1 137 146 10.1016/j.socscimed.2008.03.027 18433963

[B51] H.M. Prision Services Female Prisoners http://www.hmprisonservice.gov.uk/adviceandsupport/prison_life/femaleprisoners/Retrieved 11th February, 2010

[B52] Koffman J Higginson I Accounts of carers' satisfaction with health care at the end of life: A comparison of first generation black Caribbeans and white patients with advanced disease Palliative Medicine 2001 15 4 337 10.1191/026921601678320322 12054151

[B53] Koffman JS Higginson IJ Fit to care? A comparison of informal caregivers of first-generation Black Caribbeans and White dependants with advanced progressive disease in the UK Health & Social Care in the Community 2003 11 6 528 536 10.1046/j.1365-2524.2003.00459.x 14629584

[B54] Spruyt O Community-based palliative care for Bangladeshi patients in east London. Accounts of bereaved carers Palliative Medicine 1999 13 2 119 129 10.1191/026921699667569476 10474694

[B55] Welch L Teno J Mor V End-of-life care in black and white: Race matters for medical care of dying patients and their families Journal of the American Geriatrics Society 2005 53 7 1145 1153 10.1111/j.1532-5415.2005.53357.x 16108932

[B56] Gaffin J Hill D Penso D Opening Doors: Improving access to hospice and specialist palliative care services by members of the black and minority ethnic communities. Commentary on palliative care British Journal of Cancer 1996 74 S51 S53 PMC2149863 8782800

[B57] Koffman J The language of diversity: controversies relevant to palliative care research European J Palliative Care 2006 13 1

[B58] Koffman J Higginson IJ Religious faith and support at the end of life: a comparison of first generation black Caribbean and white populations Palliative Medicine 2002 16 6 540 541 10.1191/0269216302pm600xx 12465703

[B59] Payne S Community hospitals: an under-recognised resource for palliative care Journal of The Royal Society of Medicine 2004 97 428 431 10.1258/jrsm.97.9.428 15340022 PMC1079584

